# Application and effectiveness of *Methylobacterium symbioticum* as a biological inoculant in maize and strawberry crops

**DOI:** 10.1007/s12223-023-01078-4

**Published:** 2023-08-01

**Authors:** Rocío Torres Vera, Antonio José Bernabé García, Francisco José Carmona Álvarez, Jesús Martínez Ruiz, Félix Fernández Martín

**Affiliations:** R&D Department, Symborg SLU, Murcia, Spain

**Keywords:** Biofertiliser, Biostimulant, Maize, *Methylobacterium symbioticum*, Nitrogen-fixing bacteria, Strawberry

## Abstract

The effectiveness of *Methylobacterium symbioticum* in maize and strawberry plants was measured under different doses of nitrogen fertilisation. The biostimulant effect of the bacteria was observed in maize and strawberry plants treated with the biological inoculant under different doses of nitrogen fertiliser compared to untreated plants (control). It was found that bacteria allowed a 50 and 25% decrease in the amount of nitrogen applied in maize and strawberry crops, respectively, and the photosynthetic capacity increased compared with the control plant under all nutritional conditions. A decrease in nitrate reductase activity in inoculated maize plants indicated that the bacteria affects the metabolism of the plant. In addition, inoculated strawberry plants grown with a 25% reduction in nitrogen had a higher concentration of nitrogen in leaves than control plants under optimal nutritional conditions. Again, this indicates that *Methylobacterium symbioticum* provide an additional supply of nitrogen.

**Supplementary Information:**

The online version contains supplementary material available at 10.1007/s12223-023-01078-4.

## Introduction

Bacteria of the genus *Methylobacterium* (Patt et al. 1976) are classified as belonging to the class *Alphaproteobacteria*, the Gram-negative bacteria being characterised by a pinkish pigmentation due to the synthesis of carotenoids (Van Dien et al. 2003), mainly xanthophiles. They are rod-shaped, strictly aerobic, and can grow using compounds containing only one carbon (C1), such as methanol or methylamine (Toyama et al. 1998; Konovalova et al. 2007). *Methylobacterium* spp. can occupy different habitats, including soil, water, leaf surfaces, nodules, grains, and air (Tani et al. 2012; Wellner et al. 2012), and have been detected in more than 70 species of plants (Omer et al. 2004). There are symbiotic species, such as *Methylobacterium nodulans*, which are known to induce the formation of radical nodules in legumes of several *Crotalaria* species (Jourand et al. 2004), while the vast majority establish epiphytic or endophytic relationships (Elbeltagy et al. 2000; Wanderley Costa et al. 2012). The epiphytic species often advance along the leaf surface and invade the stomatic pores, where they can then establish endophytic bacterial communities (Kutschera 2007), which do not cause damage or form visible external structures in the host (Ardanov et al. 2012; Dourado et al. 2015).

In the phyllosphere, only about 30% of *Methylobacterium* spp. can fix atmospheric nitrogen (Madhaiyan et al. 2015). However, this atmospheric nitrogen is a great advantage for the establishment of these species in the phyllosphere, as nitrogen is often the most limiting nutrient for plant growth (Conley et al. 2009). Atmospheric nitrogen is not available for plant’s metabolism (Dourado et al. 2015), and nitrogen-fixing bacteria reduces atmospheric N_2_ to ammonium (NH_4_^+^) through the enzyme nitrogenase (*nifH* gene).

Phosphorus is the second most important element in mineral nutrition, but is not directly available for plants as it is bound to calcium, iron, or aluminium or is immobilised as a phytate (Lim et al. 2007; Agafonova et al. 2013). There are some species belonging to *Methylobacterium* spp. that can dissolve inorganic phosphates, which then become available to the plant (Agafonova et al. 2013). This is due to enzymes such as phosphatase or phytase, as well as to the secretion of organic acids, which reduce the pH of the medium (Agafonova et al. 2013; Dourado et al. 2015). Some *Methylobacterium* spp. can also protect host plants by synthesising a wide spectrum of antimicrobial molecules (Ryan et al. 2008) such as bacteriocins and organic acids, including 4-hydroxybenzoic acid (Kwak et al. 2014; Dourado et al. 2015) or by activating Induced Systemic Resistance (ISR) (Nigris et al. 2013) through the production of hydrolytic enzymes such as pectinase and cellulase (Ardanov et al. 2012). In addition, it has been observed that *Methylobacterium* spp. produce hydroxamate-type siderophores, capable of inhibiting the growth of pathogens in the plant (Sharma and Johri 2003; Andreote et al. 2008).

Due to their ability to offer plants a source of nitrogen and phosphorus, as well as to protect plants from pathogens, these microorganisms play a crucial role as *Plant Growth Promoting Bacteria* (PGPBs) (Dourado et al. 2015), which can lead to an improvement in the development of agricultural crops. Recently, a new species, *Methylobacterium symbioticum*, has been isolated from spores of the symbiont fungus species *Glomus iranicum* var. *tenuihypharum* (Pascual et al. 2020). These authors demonstrated how *Methylobacterium symbioticum* reduced the need for nitrogen chemical fertilisers, without reducing growth or the yield of rice, maize, and wine grape crops acting as a biofertiliser (Pascual et al. 2020). The yield of most of the crops treated with *Methylobacterium symbioticum* were higher than those of untreated crops, because of the ability of the bacteria to fix atmospheric nitrogen. This enzymatic activity is possible due to the presence of nitrogenase complex composed of chlorophyllide reductases and ferredoxin: protochlorophyllide reductases (ATP-dependent) (NCBI: txid2584084) that compensate for the reduction in chemical nitrogen fertilisation. Molybdenum-containing nitrogenase (encoded by *nif* genes) is the more conventional enzyme that regulates nitrogen fixation, although its central metal ion can be replaced by vanadium (encoded by *vnf* genes), this being the specific case of *Methylobacterium symbioticum* (*Methylobacterium symbioticum* 533 aa protein VUD72833.1 GI:168990407) (Sippel and Einsle 2017; Mus et al. 2018).

This new species of the genus *Methylobacterium* is postulated as an efficient biological inoculant for agriculture, so it deserves further study. Therefore, an exhaustive study of the application of *Methylobacterium symbioticum* on maize and strawberry plants was carried out in order to determine its biostimulant/biofertiliser effect on the crops under different doses of nitrogen fertilisation.

## Materials and methods

### Plant material and microorganisms used

Maize plants (*Zea mays*) of the variety EP4636 (Monsanto, San Luis, Missouri, USA) and strawberry plants (*Fragaria vesca*) of the variety Fortuna were used, and the recently isolated and described bacterium *Methylobacterium symbioticum* strain SB23 was used as inoculum (Pascual et al. 2020).

### Plant growth and treatments applied

#### Maize crop assay

The assay was carried out in a greenhouse at Symborg Experimental Farm (Los Martínez del Puerto, Murcia, Spain). The 150-L containers contained a mixture of sterile silica sand substrate, farm soil, and coconut fibre in a 1:1:1 ratio (3 plants per container). Maize seeds were sown on 17/11/2017, and the plants were harvested on 30/04/2018. The irrigation water used came from a desalination plant (Table S1), and during the assay, 130 L of water per plant were applied at a concentration of 0.1 g/L of nitrate (13 g per plant). A 3.5 kv electric pump was used to feed the water to a 32 mm secondary pipe connected to 16 mm tubes provided with 3 L/h self-compensating external drippers. The commonly used quantity of fertiliser units for this crop was provided, except in the case nitrogen, when the 0, 206, 412, and 825 fertiliser units (NFU) used, corresponding to 0, 50, 100, and 200%, respectively, of the standard nitrogen input for maize (Table S2). The nutritional needs of the plants were supplied in the irrigation system. When the plants were in stage 14 of the BBCH scale, *Methylobacterium symbioticum* was sprayed on the leaves with water at a rate of 1 × 10^5^ colony forming units (CFU) per plant, using a sprayer. Plants treated with sterile bacterial inoculum were used as control.

#### Strawberry crop assay

The experiment was carried out in a field located on the experimental plot belonging to S*ymborg Corporate* in Los Martínez del Puerto, Murcia. The plot is 8 m wide and 24 m long (192 m^2^), and the planting frame was 2 m between rows and 0.1 m between plants. The strawberry plants were provided by nurseries and sown following a quincunx pattern. They were transplanted into 1 m × 0.18 m × 0.15 m coconut fibre bags, giving a sowing density of 50,000 plants per ha. Transplantation was carried out on 02/11/2017, and the plants were harvested on 01/06/2018. The irrigation water used during the assay came from a desalination plant (Table S1), and a total of 130 L of water per plant were applied, with a concentration of 0.1 g/L of nitrate (13 g per plant). The water was supplied in exactly the same way as in the above assay, and the fertilisation units were those corresponding to strawberry plants. However, in the case of nitrogen 13, 180, 348, 515, and 683 NFU (representing 0, 25, 50, 75, and 100%, respectively) of the standard nitrogen input for strawberry crops were used (Table S2). The nutritional contribution was made through the irrigation system. The bacteria *Methylobacterium symbioticum* was applied via the leaves at 1 × 10^10^ CFU/ha. Foliar application was carried out with a spray backpack at the rate of 200 L/ha when the plant was in stages 13–14 of the BBCH scale. It was applied at an ambient temperature of 20 °C and 50% relative humidity, with no dew on the leaves. Plants treated with sterile bacterial inoculum were used as control.

### Evaluation parameters and methodology

#### Quantification of *Methylobacterium symbioticum* on leaf (CFU/g)

The CFU of *Methylobacterium symbioticum* was assessed in a leaf sample serially diluted in water. For the first dilution, 1 g of the leaf was chopped with a sterile scalpel and incubated up to 10 mL with sterile distilled water for 1 h at 160 rpm, thus favouring the extraction of bacteria from both the internal and external tissue. Serial dilutions were plated on methanol selective medium with minimal salt without a nitrogen source (Pascual et al. 2020). After 7 days of incubation at 28 °C, the colonies of *Methylobacterium symbioticum* grown in the selective medium were quantified. The CFU present in maize leaves were quantified after 2 months of growth, using 3 biological replicates per treatment. For the strawberries, the bacteria was quantified after 1, 2, 3, and 4 months of growth. Three biological replicates were used, each composed of a 50 plant leaf mixture per treatment.

#### Quantification of nitrate reductase activity

Leaf nitrate reductase (NR) activity was measured using the method described by Daniel and Curran (1981). For this purpose, after 2 months of maize plant growth, 3 biological replicates consisting of 2 technical replicates were used for each treatment studied. The strawberry crop was analysed after 2 and 3 months of growth using 3 biological replicates, each formed by the mixture of leaves from 25 plants per treatment.

#### Nitrogen content (g/100 g plant)

This parameter is an indicator of the amount of nitrogen that has accumulated in plant tissue at the time of analysis. Using the Kjeldahl method (Kjeldahl 1883), six biological replicates of each treatment were used, analysing the total nitrogen in each plant at the end of cultivation in the case of maize. Similarly, total plant nitrogen was analysed in the strawberry crops, but taking in this case 4 biological replicates, each composed of 25 plants per treatment.

#### Photosynthetic capacity of the plant

Plant photosynthetic capacity was measured using a SPAD-Chlorophyll Meter (SPAD 502-Plus, Konica Minolta, Aurora, United States). The SPAD (Soil–Plant Analysis Development) value is considered a parameter of the physiological state of the plant because it is proportional to the photosynthetic capacity of the plant and an indirect indicator of the chlorophyll content (Eke et al. 2000; Ling et al. 2011). In maize crops, after 2, 3, and 4 months of growth, the SPAD value was measured on the fourth true leaf of 6 biological replicates for each treatment. Similarly, the SPAD values were measured in strawberry plants, but using 20 biological replicates for each treatment. Each biological replicate of both tests consisted of the average value of 2 technical replicates.

#### Crop yield

After harvesting all the maize cobs, the average yield (based on the kg harvested per plant) and the yield per ha for each treatment studied were calculated. To assess the yield of the strawberry crop, 30 plants were randomly selected from each treatment and harvested throughout the growing cycle.

### Statistical analysis

All the study parameters were analysed using ANOVA, using Statgraphics Plus software for Windows 5.1 (Maugistics, Inc., USA).

## Results

### Effect of *Methylobacterium symbioticum* on the growth and development of maize crops grown under different nitrogen conditions

#### Presence of *Methylobacterium symbioticum* in the phyllosphere

Following foliar application of *Methylobacterium symbioticum*, the number of colonies in leaves of maize plants grown with different doses of nitrogen was quantified. No colonies were observed in the control plants, indicating that the assay had been properly carried out. The amount of *Methylobacterium symbioticum* quantified in treated plants 4 weeks after bacterial inoculation was between 1.27 × 10^2^ and 3.33 × 10^3^ CFU/g of leaf, but there was no linear relationship between the number of CFU counted and the amount of nitrogen provided to plants (Table 1).

**Table 1:** Quantification of *Methylobacterium symbioticum* on leaf (CFU/g) of maize plants treated and non-treated with *Methylobacterium symbioticum* and grown with different doses of nitrogen (0, 50, 100, and 200%) 4 weeks after application. Three biological replicates were used for each treatment and dose of nitrogen

	*M. symbioticum* CFU/g	
Nitrogen doses (%)	Control	*M. symbioticum* treated
0	0 ± 0	1.3 × 10^2^ ± 6.08 × 10^1^
50	0 ± 0	1.47 × 10^3^ ± 9.24 × 10^2^
100	0 ± 0	1.27 × 10^2^ ± 6.43 × 10^1^
200	0 ± 0	3.33 × 10^3^ ± 1.15 × 10^3^

#### Plant nitrate reductase activity

**Fig. 1:**
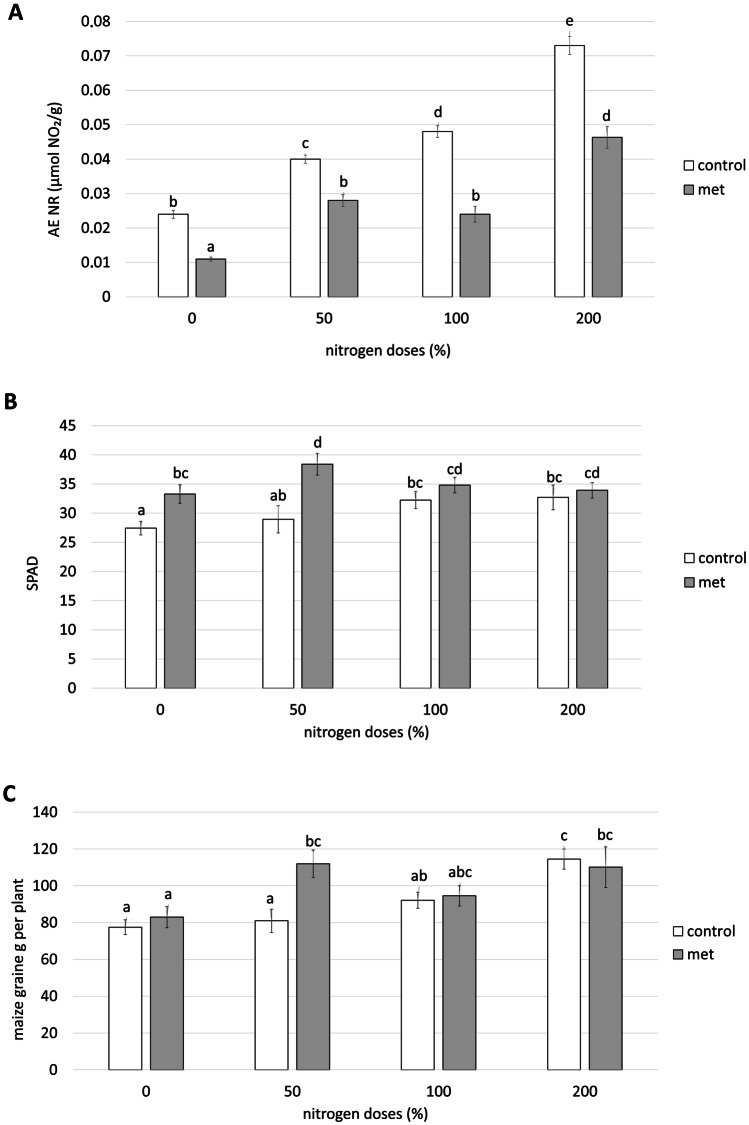
**A** Nitrate reductase activity (µmol NO_2_/g of fresh leaf after 1 h of incubation), **B** SPAD rates, and **C** grains g in maize plants treated and non-treated with *Methylobacterium symbioticum* (Met) and grown with different doses of nitrogen (0, 50, 100, and 200% of nitrogen). Simple ANOVA analysis. Different letters indicate significant differences, with *p* < 0.05 (Fisher LSD test)

There was a linear correlation between nitrogen supply and nitrate reductase activity in the control plants. In plants treated with *Methylobacterium symbioticum*, the nitrate reductase activity tended to increase as the nitrogen provided increased. However, under the same nitrogen input conditions, all the plants inoculated with *Methylobacterium symbioticum* showed significantly lower nitrate reductase activity than the control plants. More specifically, as the nitrogen dose increased, the reduction reached 56, 27, 57, and 36%, respectively, for doses representing 0, 50, 100, and 200% of nitrogen (Fig. 1A).

#### Plant photosynthetic activity

In control plants, there is a linear relationship between the SPAD values and the nitrogen dose. Photosynthetic activity was significantly higher in control plants grown with a nitrogen doses of 100 and 200%, compared with the SPAD values in the absence of this nutrient (Fig. 1B). On the other hand, the SPAD index for plants inoculated with *Methylobacterium symbioticum* was equal to or greater than that of control plants for all the nitrogen fertiliser values (Fig. 1B). When the nitrogen supply was 0%, the photosynthetic activity of treated plants was significantly higher (21%) than in the control plants in the same conditions (Fig. 1B). Similarly, with a 50% nitrogen deficiency, the SPAD value of treated plants was significantly (31%) higher than in control plants (Fig. 1B).

#### Crop yield

When the plant growth cycle had finished, the cobs were harvested and the g of maize grain generated per plant were calculated. A comparison with the yields obtained from the control plants grown in the different nutritional conditions pointed to a linear relationship between the g of maize produced and the nitrogen dose provided (Fig. 1C). On the other hand, when the yields obtained in plants inoculated with *Methylobacterium symbioticum* were compared with yields obtained from the control plants, there were only significant differences in the case of the treatment representing half the usual nitrogen supply. Indeed, the plants treated with *Methylobacterium symbioticum* generated 38.5% more grain per plant than the control plants grown under the same nutritional conditions. In addition, this treatment produced the highest yield recorded in the experiment, although the 21% increase was not significantly different from the yield of control plants grown without nitrogen deficiency (Fig. 1C).

### Effect of *Methylobacterium symbioticum* on the growth and development of strawberry crops grown under different nitrogenous conditions

#### Permanence of *Methylobacterium symbioticum* in the phyllosphere

After foliar application of *Methylobacterium symbioticum*, the number of colonies present in the leaves (intently and superficially) was quantified over a period of 90 days. In control plants, the presence of *Methylobacterium symbioticum* was not observed at any of the times analysed, indicating that no manipulation errors occurred in the experiment (Table 2). The amount *of Methylobacterium symbioticum* quantified in treated plants was between 1 × 10^3^ and 1.8 × 10^4^ CFU/g leaf, with no linear relationship between the number of CFU and the amount of nitrogen provided to plants (Table 2).

**Table 2:** Quantification of *Methylobacterium symbioticum* on leaf (CFU/g) of strawberry plants treated and non-treated with *Methylobacterium symbioticum* and grown with different doses of nitrogen (0, 25, 50, and 100%) after 1, 30, 60, and 90 days post inoculation (dpi). Three biological replicates were used for each replicates for each treatment and applied nitrogen

		*M. symbioticum* CFU/g			
Treatment	Nitrogen doses (%)	1 dpi	30 dpi	60 dpi	90 dpi
Control	0	0 ± 0	0 ± 0	0 ± 0	0 ± 0
	25	0 ± 0	0 ± 0	0 ± 0	0 ± 0
	50	0 ± 0	0 ± 0	0 ± 0	0 ± 0
	75	0 ± 0	0 ± 0	0 ± 0	0 ± 0
	100	0 ± 0	0 ± 0	0 ± 0	0 ± 0
*M. symbioticum*	0	9.33 × 10^3^ ± 1.15 × 10^3^	2 × 10^3^ ± 8.66 × 10^2^	2 × 10^3^ ± 0	2.5 × 10^3^ ± 5 × 10^2^
	25	2.33 × 10^3^ ± 1.53 × 10^3^	1.1 × 10^3^ ± 3.16 × 10^2^	8 × 10^3^ ± 2.65 × 10^3^	8 × 10^3^ ± 2.65 × 10^3^
	50	2 × 10^3^ ± 1.73 × 10^3^	1.07 × 10^3^ ± 1.15 × 10^2^	6.47 × 10^3^ ± 1.5 × 10^3^	5 × 10^3^ ± 2.09 × 10^3^
	75	1.80 × 10^4^ ± 7.21 × 10^3^	3 × 10^3^ ± 5 × 10^2^	1.03 × 10^3^ ± 5.77 × 10^1^	3.03 × 10^3^ ± 7.23 × 10^2^
	100	2.60 × 10^3^ ± 1.15 × 10^3^	1 × 10^3^ ± 0	1.1 × 10^3^ ± 1 × 10^2^	1.03 × 10^3^ ± 5.77 × 10^1^

#### Plant nitrate reductase activity

**Fig. 2:**
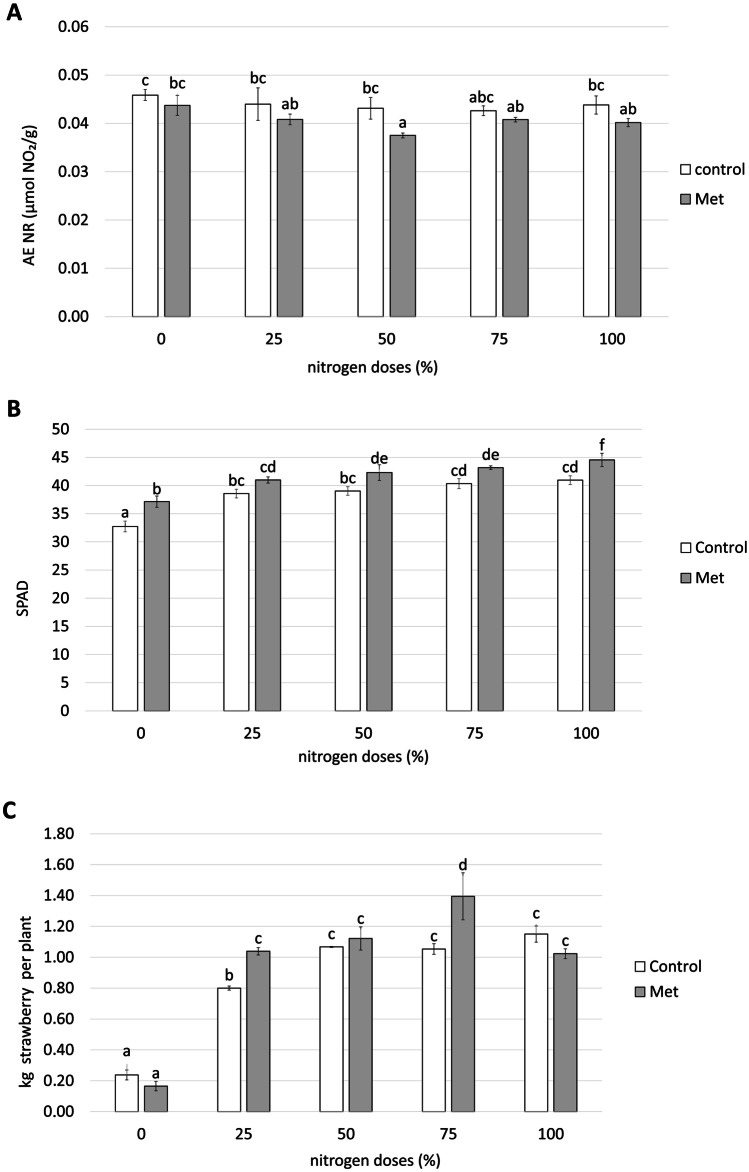
**A** Nitrate reductase activity (µmol NO_2_/g of fresh leaf after 1 h of incubation), **B** SPAD rates, and **C** fruit kg in strawberry plants treated and non-treated with *Methylobacterium symbioticum* (Met) and grown with different doses of nitrogen (0, 25, 50, and 100% of nitrogen). Simple ANOVA analysis. Different letters indicate significant differences, with *p* < 0.05 (Fisher’s LSD test)

No dose-dependent pattern in nitrate reductase activity was detected in control plants according to the dose of nitrogen used (Fig. 2A). In contrast, nitrate reductase activity of plants treated with *Methylobacterium symbioticum* had lower levels of nitrate reductase activity than control plants for all the nitrogen doses studied. The lowest value in treated plants was observed with 50% nitrogen, when the nitrate reductase activity detected was 13% lower than in control plants in the same conditions and 14.4% lower than in the control plants under normal nitrogen conditions. Both results were statistically significant (Fig. 2A).

#### Plant photosynthetic activity

There was a gradual increase in photosynthetic activity as the nitrogen dose increased, all values being significantly higher than the SPAD values of control plants in the absence of the nutrient. However, the SPAD index for plants treated with *Methylobacterium symbioticum* was higher than in control plants for all nitrogen doses used (Fig. 2B). Plants treated with *Methylobacterium symbioticum* in conditions that represented 25, 50, 75, and 100% of the usual nitrogen supply showed values that were equal to or greater than those of control plants with 100% nitrogen. In addition, at 100% nitrogen, the treated plants had 8.8% more photosynthetic activity than control plants at the usual nitrogen dose, the differences being significant.

#### Crop yield

During the strawberry growing cycle, strawberries from 100 plants from each treatment and nitrogen dose were harvested weekly. A comparison of the yield of the control plants amongst themselves in the different nutritional conditions pointed to a linear relationship between the kg of strawberries harvested and the nitrogen dose provided (Fig. 2C). An analysis of the results obtained in plants inoculated with *Methylobacterium symbioticum* identified a significant increase in yield of 29.9% and 32.6% under conditions of 25 and 75% nitrogen compared to control plants under the same conditions (Fig. 2C). In addition, plants treated with the 75% dose of nitrogen produced the highest amount of kg observed in the experiment, the 21.3% increase being statistically significant compared with the yield of non-nitrogen deficient control plants (Fig. 2C). When the nitrogen supply was 0, 50, and 100%, the treated plants showed no significant differences from their controls with the same nitrogen dose (Fig. 2C).

#### Assimilation of nitrogen in plant and its relationship with the yield and nitrogen dose provided

In the final stage of growth of the strawberry plants, it was observed that the plants assimilated less nitrogen than provided in all the treatments, except in the treatment involving no added nitrogen (Table 3). In control plants, the assimilation levels of nitrogen by the plant was directly related with the gradual increase in nitrogen supply. In addition, under optimal nitrogen conditions (100%), control plants showed the maximum values for yield and nitrogen assimilation. On the other hand, it was found that with a dose of 25 and 50% nitrogen, the yield of plants treated with *Methylobacterium symbioticum* did not show significant differences compared to control plants under optimal nutritional conditions. In addition, plants treated with *Methylobacterium symbioticum* and receiving 75% nitrogen gave the highest yields and showed the greatest level of nitrogen assimilation of all the plants and treatments of the assay, with a 21.7% higher yield and 11 kg/ha more assimilated nitrogen than control plants under optimal conditions. Surprisingly with a contribution of 100% nitrogen, plants inoculated with *Methylobacterium symbioticum* the yield and nitrogen uptake/assimilation values fell by 27.1 and 9.9%, respectively, compared to the inoculated plants with a contribution of 75% nitrogen. However, this result does not present significant differences with respect to the control plants under optimal nutritional conditions (Table 3).

**Table 3:** Dose of nitrogen provided (NFU/ha), assimilated nitrogen (kg/ha), and yield (kg per plant) of strawberry plants treated and non-treated with *Methylobacterium symbioticum* and grown with different doses of nitrogen (0, 25, 50, and 100% of nitrogen). Simple ANOVA analysis. Different letters indicate significant differences, with *p* < 0.05 (Fisher’s LSD test). One hundred biological replicates were used for each treatment and applied nitrogen

	Nitrogen doses		Assimilated nitrogen	Yield
Treatment	%	NFU/ha	kg/ha	kg per plant
Control	0	13	14	0.24 a
	25	180	104	0.8 b
	50	348	295	1.07 c
	75	515	312	1.05 c
	100	683	313	1.15 c
*M. symbioticum*	0	13	14	0.17 a
	25	180	115	1.04 c
	50	348	292	1.12 c
	75	515	324	1.4 d
	100	683	292	1.02 c

## Discussion

The continuous addition of nitrogen fertilisers obtained by chemical synthesis severely affects ecosystems (Mahanty et al. 2016), making biofertilisers based on beneficial microorganisms an attractive alternative. Pascual and co-workers demonstrated how the application of the nitrogen-fixing bacterium *Methylobacterium symbioticum* in various crops permitted conventional nitrogen fertilisation to be reduced, while providing equal or better yields than those obtained with conventional agricultural treatments (Pascual et al. 2020).

Some authors hypothesise that there is a degree of bacterium-plant specificity, in which both groups co-evolve (Romanovskaya et al. 1999; Dourado et al. 2012). Due to this possible specificity, we studied the interaction of *Methylobacterium symbioticum* in two very different crops, maize and strawberries. Parameters related to growth and development and nitrogen metabolism of plants grown under different nitrogen input conditions were analysed.

After foliar application, we observed that, in both maize and strawberry plants inoculated with *Methylobacterium symbioticum*, nitrogen supply did not affect the survival of *Methylobacterium symbioticum* in the phyllosphere. Differences were detected between the amount of *Methylobacterium symbioticum* in maize and strawberry plants, but it is known that the population density of endophytic bacteria varies greatly with the type of plant and bacterial strain, as well as the environmental growing conditions (Pillay and Nowak 1997). In addition, a constant population of *Methylobacterium symbioticum* was observed in strawberry crops throughout the crop development cycle. With this result, we observe that the application time of *Methylobacterium symbioticum* application can be adapted to the needs of the crop, allowing the possible benefits to be prolonged. Numerous studies suggest that the success of *Methylobacterium* strains in the colonisation of the phyllosphere is due to its ability to metabolise methanol emitted through plant stomata and use it as a source of carbon and energy (Sy et al. 2005). However, these strains are capable of offering carbon to the plant, thanks to their ability to carry out anoxygenic photosynthesis due to bacteriophytochromes present in their metabolism (Giraud and Vermeglio 2008). Specific bacteriochlorophylls (chlorophyllide reductases; NCBI:txid2584084) have been described in *Methylobacterium symbioticum* that regulates this process, mainly using (C1) compounds such as methanol and formaldehydes as carbon source (Sy et al. 2005; Kutschera 2007).

Due to the diazotrophic nature of *Methylobacterium symbioticum*, we studied whether there was a metabolic relationship between bacterial inoculation and the plant nitrogen requirements. Nitrate (NO_3_^−^) is considered as one of the main sources of nitrogen available to plants (Schrader and Hageman 1967). In order to assimilate it, nitrate must be converted to ammonium (NH_4_^+^) by the plant through the activity of the enzymes, nitrate reductase, and nitrite reductase (Kaiser and Huber 1994; Borsani et al. 1999; Wang et al. 2014). This biochemical process is highly regulated, so nitrate reductase activity can be used as an indicator of alterations in the nitrogen metabolism of the plant. For all the nitrogen percentages used, nitrate reductase activity was lower in plants inoculated with *Methylobacterium symbioticum* than in both maize and strawberry control plants. It has previously been described how high concentrations of ammonium in the plant have an inhibitory effect on nitrate reductase activity, since its activity is not essential (Ohyama 2010). Our results seem to point to high bacterial activity during the fixation of atmospheric nitrogen, which is regulated by a reduction in reductase activity in the plant. This coincides with the observations of Pascual and co-workers (2020) in maize plants treated with *Methylobacterium symbioticum*, in which plant enzymatic activity was seen to decrease. Despite the decrease in nitrate reductase activity in treated plants, an inversely proportional decrease was not shown for all applied nitrogen doses. This may be due to the fact that the nitrate reductase activity is regulated not only by the amount of ammonium in the plant, which is partly supplied by *Methylobacterium symbioticum*, but also depends on the C/N ratio present in the plant, the oxygen conditions, the presence or absence of vanadium, and lighting conditions (Giraud and Vermeglio 2008; Sippel and Einsle 2017; Mus et al. 2018).

The values of SPAD are proportional to the amount of chlorophyll present in the leaves and, therefore, to their photosynthetic activity (Eke et al. 2000; Ling et al. 2011). The results obtained for both maize and strawberry control plants pointed to an increase in the photosynthetic activity of all treatments compared with the results obtained without additional nitrogen. This result confirms that the different nitrogenous treatments are detected by plants, which assimilate the nitrogen, since its presence is related with greater photosynthetic activity. Plants inoculated with *Methylobacterium symbioticum* had similar or higher values of SPAD in maize than those found in control plants under the same growing conditions, while the values were always higher in strawberry, confirming the positive effect that *Methylobacterium symbioticum* have on the photosynthetic capacity of the plant. In addition, inoculation allowed the amount of nitrogen added to be reduced by 100% in maize and 75% in strawberry, with no quantitative loss of SPAD compared with control plants in optimal conditions. This shows that, in conditions of nitrogen stress, the plant can promote its symbiosis with *Methylobacterium symbioticum.* This is consistent with studies by Cervantes et al. (2004) and Madhaiyan et al. (2015), who recorded higher photosynthetic activity in plants inoculated with several species of *Methylobacterium* compared with non-inoculated plant. In molecular studies, multiple strains of *Methylobacterium* spp. were seen to contain the genes involved in photosynthesis, such as those that encode light-harvesting complexes and the biosynthesis of bacteriochlorophylls and carotenoids (Marx et al. 2012).

Our results suggest that treatment with *Methylobacterium symbioticum* could supply 50% of the conventional nitrogen supplied to the maize crop, accompanied by a 21% increase in yield. Strawberry plants inoculated with *Methylobacterium symbioticum* behaved in the same way, the bacteria compensating for a 50 and 75% nitrogen deficiency, and even improving yield by 21% when the deficiency was 25% of the conventional nitrogen supply. Improved performance in plants treated with *Methylobacterium symbioticum* may result from ammonium uptake provided by the microorganism through nitrogen fixation, which would result in a saving of energy for the plant. These results in strawberry are in agreement with the values of photosynthetic activity detected for the plants treated in the 3 doses of nitrogen mentioned, since they were similar to the values of the control plants under optimal conditions. However, only the plants treated and grown with a 25% nitrogen deficiency showed a significant increase in yield and in the amount of nitrogen in the leaf compared to control plants. This result may be related to a high leaching of nitrogen in the soil caused by the continuous irrigation applied to the strawberry crop, since a higher yield would be expected for the treated plants grown with a deficiency of 50% compared to those grown with deficiency of 75%. The biofertilising effect of *Methylobacterium symbioticum* has also been described in other species of the genus such as *Methylobacterium radiotolerans* in peanuts *(Arachis hypogaea*) and *Methylobacterium extorquens* in strawberry crops amongst others (Abanda-Nkpwatt et al. 2006; Priya et al. 2019). Some studies have shown that nitrogen supplementation caused by the application of the species *Methylobacterium suomiense* and *Methylobacterium oryzae* has a biofertilising effect on red pepper crops (*Capsicum annuum*). This effect may be derived from increased activity of the bacterial enzyme nitrogenase, through a Fe^+3^/Mo^+2^-dependent nitrogenase system (Shah et al. 1984). Madhaiyan and co-workers (2015) observed an increase in this activity in jatropha (*Jatropha curcas*) plants inoculated with *Methylobacterium radiotolerans* and which had high yields and improved plant growth and development. In contrast, in strawberry, there was no greater assimilation of nitrogen or increase in yield in plants inoculated at the 100% nitrogen dose. Similarly, the yield of maize plants inoculated with *Methylobacterium symbioticum* was unaltered under optimal conditions, which shows that the biofertilising effect may depend on the existence of abiotic stress in the plant.

In addition to providing ammonium to the plant, it has been described how *Methylobacterium* spp. can regulate plant growth by producing hormones such as auxins or cytokinins. These hormones are key in vegetative growth (Seok et al. 2006; Yim et al. 2010), and the induction of its synthesis demonstrates the biostimulant effect of *Methylobacterium* spp. when interacting with the plant. Also, it has been suggested that some strains of *Methylobacterium* synthesise vitamin B_12_, inducing the plant development (Seok et al. 2006).

## Conclusion

To summarise, the data shows the ability of *Methylobacterium symbioticum* to fix atmospheric nitrogen and support the hypothesis that, for a *Methylobacterium*-plant association, nitrogen is transferred from the bacteria to the plant, which thus receives the nitrogenous input it needs. However, the results also point to the need for specific studies to be conducted for each crop in order to ascertain the nutritional conditions in which *Methylobacterium symbioticum* fulfils its maximum potential as a biofertiliser, just as we have done in this work for maize and strawberry crops.

### Supplementary Information

Below is the link to the electronic supplementary material.

Supplementary file1 (DOCX 16 KB)
