# Supplementary material for: Application and effectiveness of Methylobacterium symbioticum as a biological inoculant in maize and strawberry crops
Source: Folia Microbiol (Praha). 2023 Aug 1;69(1):121–31. doi: 10.1007/s12223-023-01078-4 (PMC10876812; doi:10.1007/s12223-023-01078-4)
Supplement: Supplementary file 1 — Supplementary file1 (DOCX 16 KB) [file 12223_2023_1078_MOESM1_ESM.docx]

**SUPPLEMENTARY INFORMATION**

**Table S1**. Main physical-chemical properties of the water used for irrigation supplied by a desalinating plant.

| **Parameters** | **Results** |
| --- | --- |
| pH | 7.86 |
| EC (dSm−1) | 0.96 |
| Total dissolved solids (g/l) | 0.507 |
| Chlorine anions (g/l) | 0.245 |
| Sulphate anions (g/l) | 0.0157 |
| Hydroxide anions (g/l) | 0.0001 |
| Carbonate anions (g/l) | 0.074 |
| Nitrate anions (g/l) | 0.101 |
| Soluble phosphorous anions (g/l) | 0.0005 |
| Calcium cations (g/l) | 0.0202 |
| Magnesium cations (g/l) | 0.0084 |
| Sodium cations (g/l) | 0.134 |
| Potassium cations (g/l) | 0.01 |
| Ammonium cations (g/l) | 0.00077 |
| Boron micronutrients (g/l) | 0.99 |
| Iron micronutrients (g/l) | 0.05 |
| Manganese micronutrients (g/l) | 0.01 |
| Copper micronutrients (g/l) | 0.01 |
| Zinc micronutrients (g/l) | 0.01 |

**Table S2**. Nutrient solution used for each of the nitrogen (N) treatments studied.

|  | **Maize treatment** | | | | **Strawberry treatment** | | | | |
| --- | --- | --- | --- | --- | --- | --- | --- | --- | --- |
|  | **Nitrogen doses (%)** | | | | **Nitrogen doses (%)** | | | | |
| **Nutrients** | **0** | **50** | **100** | **200** | **0** | **25** | **50** | **75** | **100** |
| Phosphoric acid 75 % (cc/m^3^) | 6 | 6 | 6 | 6 | 15 | 15 | 15 | 15 | 15 |
| Potassium sulphate (g/m^3^) | 1 | 1 | 1 | 1 | 300 | 300 | 300 | 300 | 300 |
| Magnesium sulphate (g/m^3^) | 12 | 12 | 12 | 12 | 325 | 325 | 325 | 325 | 325 |
| Micronutrients (g/m^3^) | 22 | 22 | 22 | 22 | 22 | 22 | 22 | 22 | 22 |
| Calcium chloride (g/m^3^) | 11 |  |  |  | 393 | 222 | 52 |  |  |
| Calcium nitrate (g/m^3^) |  | 14 | 14 | 14 |  | 241 | 482 | 556 | 741 |
| Ammonium nitrate (g/m^3^) |  | 37 | 88 | 190 |  |  |  | 78 | 104 |
| Potassium Mono Phosphate (g/m^3^) | 20 | 20 | 20 | 20 | 42 | 42 | 42 | 42 | 42 |
